# Dexamethasone Inhibits the Growth of B‐Lymphoma Cells by Downregulating DOT1L

**DOI:** 10.1002/cnr2.2150

**Published:** 2024-09-22

**Authors:** Yuting Wang, Nan Zhang, Weilong Shang, Huagang Peng, Zhen Hu, Yi Yang, Li Tan, Li Zhang, Fengtian He, Xiancai Rao

**Affiliations:** ^1^ Department of Microbiology, College of Basic Medical Sciences, Key Laboratory of Microbial Engineering Under the Educational Committee in Chongqing Army Medical University Chongqing China; ^2^ Department of Hematology People's Liberation Army the General Hospital of Western Theater Command Chengdu China; ^3^ Department of Oncology Xiangya Hospital, Central South University Changsha China; ^4^ Department of Biochemistry and Molecular Biology College of Basic Medical Sciences, Army Medical University Chongqing China

**Keywords:** dexamethasone, DOT1L, gene regulation, glucocorticoid receptor

## Abstract

**Background:**

Dexamethasone (Dex), a synthetic glucocorticoid that acts by binding to the glucocorticoid receptor (GR), has been widely applied to treat leukemia and lymphoma; however, the precise mechanism underlying Dex action is still not well elucidated. DOT1L, a histone H3‐lysine79 (H3K79) methyltransferase, has been linked to multiple cancer types, particularly mixed lineage leukemia (MLL) gene rearranged leukemia, but its contribution to lymphoma is yet to be delineated. Analysis from the TCGA database displayed that DOT1L was highly expressed in lymphoma and leukemia.

**Results:**

We initially demonstrated that DOT1L served as a new target gene controlled by GR, and the downregulation of DOT1L was critical for the killing of B‐lymphoma cells by Dex. Further study revealed that Dex had no impact on the transcriptional activity of the DOT1L promoter, rather it reduced the mRNA level of DOT1L at the posttranscriptional level. In addition, knockdown of DOT1L remarkably inhibited the B‐lymphoma cell growth.

**Conclusions:**

Overall, our findings indicated that DOT1L may serve as a potential drug target and a promising biomarker of Dex sensitivity when it comes to treating B lymphoma.

## 1 Introduction

Glucocorticoid is widely used due to its effective anti‐inflammatory and immunosuppressing effect [1]. Dexamethasone (Dex), a classical glucocorticoid, is recognized as one of the standard therapeutics in hematological malignancies including leukemia and lymphoma, and the clinical effect is much better than other glucocorticoids [2, 3, 4]. Dex exerts its antitumor effects primarily by binding to and subsequently activating glucocorticoid receptor (GR) [5, 6]. After translocating into the nuclear region, activated GR commonly regulates the expression of its target genes via binding to the glucocorticoid response element (GRE) [7, 8]. Although the function of GR has been studied for decades, its precise antitumor mechanism has not been clearly clarified yet.

DOT1L (disruptor of telomere silencing 1 like) is the sole methyltransferase that carries out the methylation of histone H3 at lysine 79 [9]. Previous research has reported that DOT1L is linked to multiple biological processes, such as telomere silencing, gene expression regulation, cell aging, and DNA damage response [10, 11, 12]. Recently, it has been proved that DOT1L has a crucial role in the initiation and progression of various tumor types, such as lung cancer, breast cancer, ovarian cancer, renal clear cell carcinoma, and neuroblastoma [13, 14, 15, 16]. Notably, DOT1L is strongly correlated with MLL‐rearranged leukemia [17]. DOT1L leads to aberrant H3K79 methylation that contributes to the overexpression of MLL target oncogenes such as *MEIS1* and *HOXA9*. Therefore, DOT1L is considered as a new therapeutic target against MLL‐rearranged leukemia and the corresponding inhibitors are investigated in clinical trials [18, 19, 20]. However, it remains elusive whether DOT1L is linked to B‐lymphoma cell and whether DOT1L can be regulated by Dex.

Our work shows that DOT1L plays an oncogenic role in B‐lymphoma cells and Dex downregulates DOT1L via GR activation. Besides, Dex decreases DOT1L expression in Dex‐sensitive B‐lymphoma cells and MLL‐rearranged leukemia cells, but not in Dex‐insensitive acute monocytic leukemia cells, suggesting that DOT1L may be an underlying novel indicator of Dex sensitivity against hematological malignancies.

## 2 Materials and Methods

### 2.1 Reagents

Dex, RU486, and actinomycin D (Act D) were obtained from Sigma‐Aldrich (St Louis, USA). siRNAs for DOT1L and negative control were synthesized by Invitrogen; the sequences for DOT1L siRNA included siRNA‐1, 5′‐CGCGAGUUCAGGAAGUGGAUGAAAU‐3′; siRNA‐2, 5′‐CGAUAAACAUCACGAUGCUGCUCAU‐3′; and siRNA‐3, 5′‐CGCUGCCGGUCUACGAUAAACAUCA‐3′. Dual‐luciferase reporter system was bought from Promega (Madison, USA). Primary antibodies against H3K79me2 and H3 were purchased from Cell Signaling (Danvers, MA) and Thermo Scientific (Rockford, USA), respectively, and the secondary antibodies were bought from Zhongshan Biotechnology.

### 2.2 Cell Culture

Human cell lines Raji, Daudi, Namalwa, JeKo‐1, THP‐1, Jurkat, MV4‐11, and HEK‐293 were acquired from the American Type Culture Collection (ATCC) and then cultivated in a humidified incubator set to 37°C, 5% CO_2_. Raji, Daudi, Namalwa, JeKo‐1, THP‐1, and Jurkat cells were grown in RPMI‐1640 medium (Gibco) with 10% fetal bovine serum (FBS). MV4‐11 and HEK‐293 cells were grown in IMDM and DMEM media with 10% FBS respectively.

### 2.3 Western Blot Analysis

Histones of each human cell line were prepared by the EpiQuik Total Histone Extraction Kit, and the protein concentrations were calculated using the BCA protein assay kit. Then 3 μg histones were loaded on 15% sodium dodecyl sulfate‐polyacrylamide gel electrophoresis (SDS‐PAGE) and following transferred to polyvinylidene fluoride (PVDF) membranes (Millipore). After subsequent blocking with 5% fat‐free dry milk for 2 h, the membranes were probed overnight at 4°C with antibodies against H3K79me2 and H3, followed by the relevant horseradish peroxidase‐conjugated secondary antibodies (Zhongshan Biotechnology, China). The Supersignal West Dura Extended Duration Substrate was utilized for signal detection.

### 2.4 Cell Viability Assay

Cells were cultivated overnight in 96‐well plates at a density of 2 × 10^3^ cells/well, then conducted with Dex or DMSO in the presence or absence of RU486. After that, the OD value at 450 nm was obtained by Cell Counting Kit‐8 (CCK‐8) (Dojindo laboratories, Japan). The results were normalized against the OD_450_ values of the control. Every experiment in duplicate was conducted at least three times.

### 2.5 Quantitative Real‐Time PCR

Total RNA from cells was extracted by Trizol reagent (Invitrogen, USA), and 1000 ng of total RNA was reverse transcribed into cDNA utilizing PrimeScript RT Master Mix (Takara Dalian, China). Quantitative real‐time PCR (qPCR) was conducted in triplicate with SYBR Select Master Mix (Applied Biosystems, USA), with β‐actin serving as the control. The qPCR experiment was performed using the ABI Prism 7500 detection system. Finally, we used the 2^−ΔΔCt^ method to calculate the relative mRNA levels of the target genes. The primers are provided in Table 1.

**TABLE 1:** Primers for qPCR used in this study.

Gene	Primer sequence (5′ → 3′)	Product size (bp)
*DOT1L* (human)	Forward: AGGTAACTAGGATTTCTACCTC	199
	Reverse: CTATCGACAGTACAAACTGG	
*β‐Actin* (human)	Forward: CGAGGCCCCCCTGAAC	562
	Reverse: GCCAGAGGCGTACAGGGATA	
*Meis1* (human)	Forward: CCCTGGAATGCCAATGTCA	89
	Reverse: GAGCGTGAATGTCCATGACTTG	

### 2.6 Transient Transfection

Cells were, respectively, cultivated in 48‐well plates overnight. Plasmids were transfected into HEK293 cells by lipofectamine 3000 (Invitrogen), and siRNAs were transfected into Raji, MV4‐11, and Jurkat cells by SG Cell Line 4D‐Nucleofector X Kit (Lonza). The experiments were performed by three independent biological trail.

### 2.7 Dual‐Luciferase Reporter Assays

Putative GREs in the human *DOT1L* promoter region (−2800 to +200 bp) were predicted using NUBIScan, an online Algorithm. Then, the *DOT1L* gene promoter region (−1800 to +200 bp) which including GREs was amplified with PCR and cloned into a pGL3‐basic vector (Promega), and the recombinant reporter plasmids were defined as pGL3‐DOT1L. For reporter assays, the pGL3‐DOT1L or pGL3‐basic plasmids were separately co‐transfected with pRL‐TK (Promega) expressing Renilla luciferase into HEK‐293 cells using lipofectamine 3000. After incubating for 18 h, Dex was added to the medium with or without RU486 for 6 h. Then, cells were collected and measured using the Dual‐Luciferase Assay System (Promega). To adjust the differences in the above experiment, pRL‐TK was co‐transfected as an internal control. Every assay in duplicate was done at least three times.

### 2.8 Statistical Analysis

The data were exhibited as means ± standard deviation. Student's *t*‐test and one‐way ANOVA was applied to compare two groups or multiple groups, respectively, to calculate the statistical significance (*p* value) for all data using Prism 6.0 (GraphPad). In all cases, “ns” indicated no significance, whereas *p < 0.05 was regarded as statistically significant.

## 3 Results

### 3.1 DOT1L Is Highly Expressed in B‐Lymphoma Cells

**FIGURE 1:**
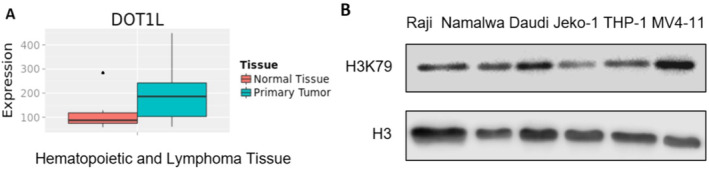
DOT1L is highly expressed in B‐lymphoma cells. (A) The relative expression of *DOT1L* between hematological malignancies and normal tissues was analyzed by the TCGA database. (B) The basal protein level of DOT1L effector H3K79me2 was examined in several B‐lymphoma cells by Western blot. H3 served as loading controls.

To investigate the expression difference of DOT1L between normal tissues and hematological malignancies, the TCGA database was used. As demonstrated in Figure 1A, DOT1L expression was higher in hematopoietic and lymphoid tumors than in normal tissues. Moreover, the basal expression of H3K79 (the downstream effector of DOT1L) was examined in several B‐lymphoma cells. The MLL‐rearranged cell line MV4‐11 was known to express a high level of DOT1L/H3K79 and was used as a positive control [18]. As illustrated in Figure 1B, H3K79 was relatively highly expressed in three Burkitt's lymphoma cells (Raji, Namalwa, and Daudi), but not the acute monocytic leukemia cell (THP‐1) or the mantle cell lymphoma cell (Jeko‐1). In addition, we analyzed the DOT1L mRNA expression level by the Human Protein Atlas Dataset. The result showed that the DOT1L mRNA expression level was relatively high in lymphoma (Figure S1A), and it was the highest in the Raji cell as for the above six cell lines (Figure S1B), which could partly explain why the Raji cell line was used in the study. Taken together, the above results indicated that DOT1L might contribute to the initiation and development of B lymphoma.

### 3.2 Silencing *DOT1L* Significantly Inhibits the Growth of B‐Lymphoma Cells

**FIGURE 2:**
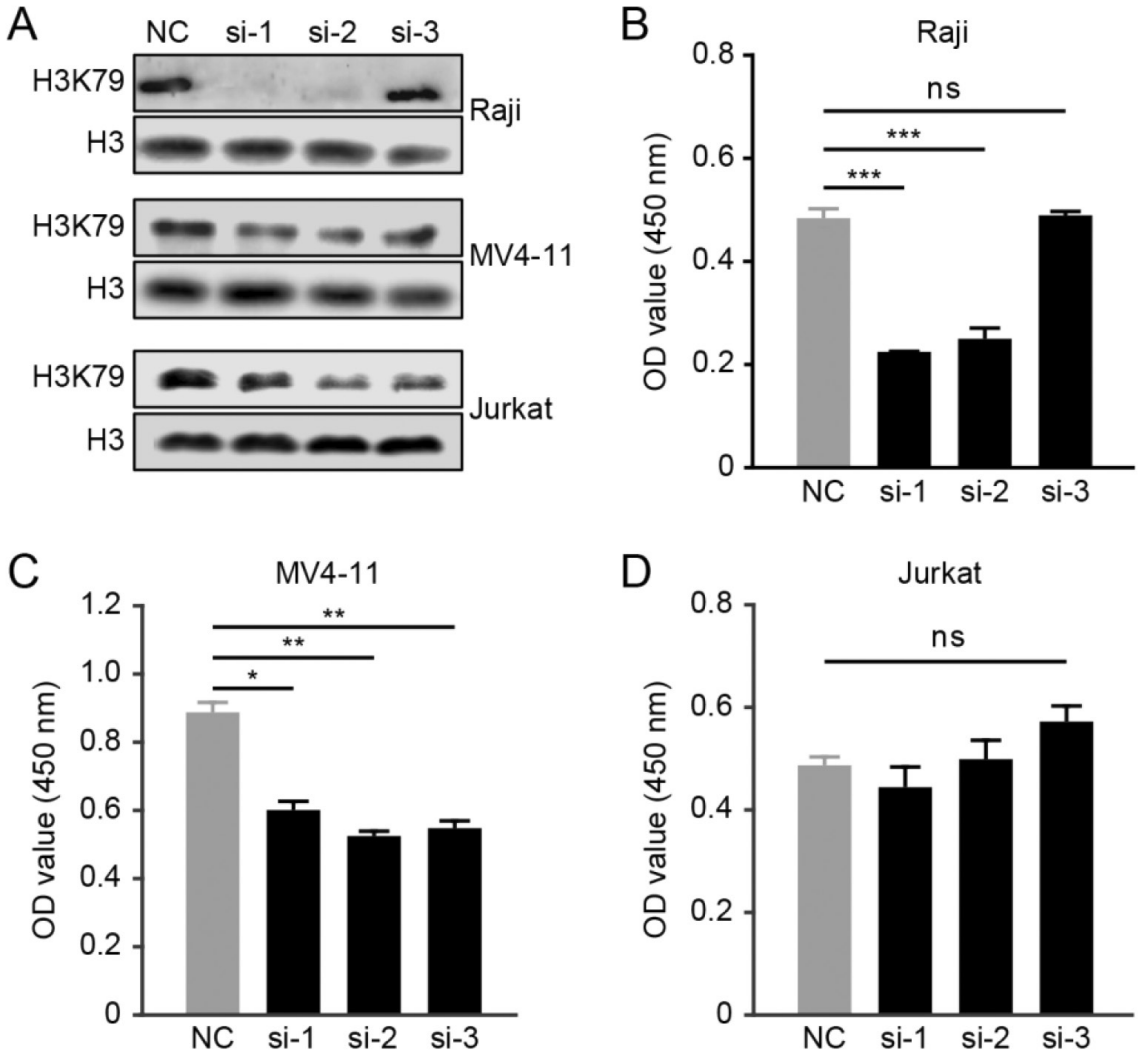
Silencing *DOT1L* hinders the growth of B‐lymphoma cells. (A) The silencing effect of siRNA against *DOT1L* was examined. (B–D) After transfection with three independent siRNAs against *DOT1L* or the control NC siRNA, Raji, MV4‐11, and Jurkat cells were incubated for 96 h and CCK‐8 assay was performed to assess the cell viability (ns, no significance; *p < 0.05; **p < 0.01; ***p < 0.001).

To examine the character of DOT1L in B‐lymphoma cells, Raji, MV4‐11, and Jurkat (the acute lymphoblastic leukemia cell line) cells were transfected with siRNA targeting DOT1L, respectively. The silencing efficiency of DOT1L was indicated by the reduction of H3K79 (Figure 2A). It has been reported that *DOT1L* is an important oncogene in the MLL‐rearranged cell line MV4‐11, a potent small‐molecule DOT1L inhibitor named EPZ000477, caused the selective killing of MV4‐11 cells rather than Jurkat cells [18]; therefore, we took MV4‐11 and Jurkat as the positive control and the negative control, respectively. Knockdown of *DOT1L* remarkably attenuated the viability of Raji (Figure 2B), whereas the viability of Jurkat cells was not affected. These data revealed that *DOT1L* also played an oncogenic role in B lymphoma.

### 3.3 GR Mediates the Downregulation of *DOT1L* and Its Target Gene by Dex in B‐Lymphoma Cells

**FIGURE 3:**
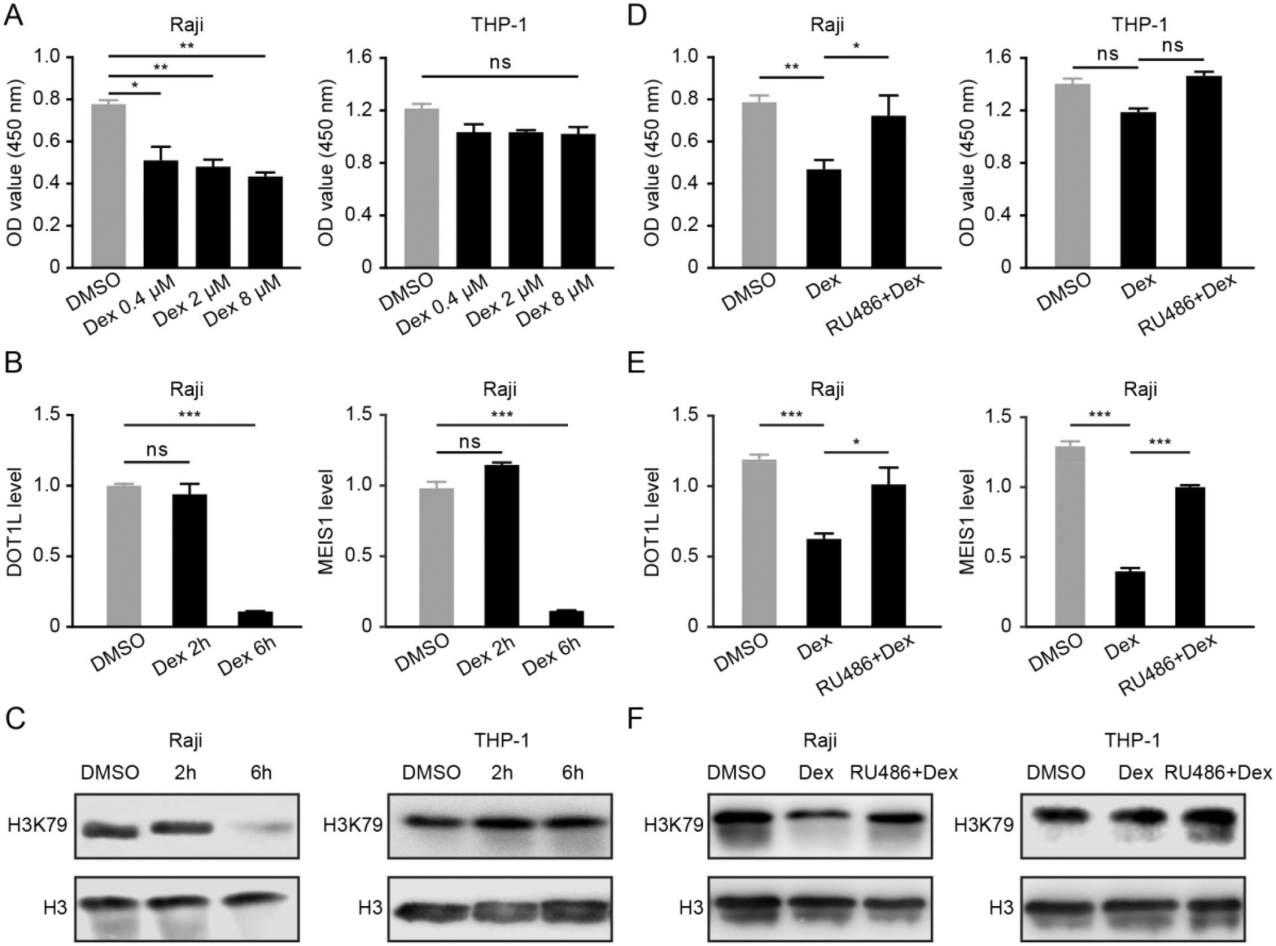
Dex downregulates DOT1L and its target gene via GR in B‐lymphoma cells. (A) Raji and THP‐1 cells were incubated with incremental concentrations of Dex, and then the CCK‐8 assay was taken to evaluate the cell viability. (B) Raji cells were incubated with 0.4 μM Dex, following the mRNA levels of DOT1L and its target gene MEIS1 were detected by qPCR. (C) Raji and THP‐1 cells were conducted with 0.4 μM Dex, then Western blot was applied to detect the protein levels of the DOT1L effector H3K79me2, and H3 served as the loading control. (D) After pretreatment with RU486 for 30 min, Raji and THP‐1 cells were incubated with Dex, and then the CCK‐8 assay was taken to evaluate the cell viability. (E) After pretreatment with 4 μM of RU486 for 30 min, Raji cells were conducted with Dex, afterwards the mRNA levels of DOT1L and its target gene MEIS1 were detected by qPCR. (F) After pretreatment with 4 μM of RU486 for 30 min, Raji and THP‐1 cells were conducted with 0.4 μM Dex, then the DOT1L effector H3K79me2 was measured by Western blot.

Dex suppressed the viability of Raji cells with an apparent concentration‐dependent effect (Figure 3A). However, Dex did not inhibit the growth of the acute lymphoblastic leukemia cell line THP‐1 even at a high concentration (8 μM), so we took the Dex‐insensitive THP‐1 cell as negative control. Moreover, Dex dramatically reduced the mRNA level of *DOT1L* and its target gene *MEIS1* in Raji cells (Figure 3B). In addition, Dex remarkably downregulated the level of H3K79 in Dex‐sensitive Raji cells but not in the Dex‐insensitive THP‐1 cells (Figure 3C). Pretreatment with GR antagonist RU486 significantly mitigated the Dex‐induced suppression of cell growth, the mRNA levels of *DOT1L* and *MEIS1*, and protein levels of H3K79 in Raji cells (Figure 3D–F). Together, Dex could downregulate *DOT1L* and its target gene in a GR‐dependent manner for B‐lymphoma cells.

### 3.4 Dex Reduces the mRNA Level of *DOT1L* at the Posttranscriptional Level

**FIGURE 4:**
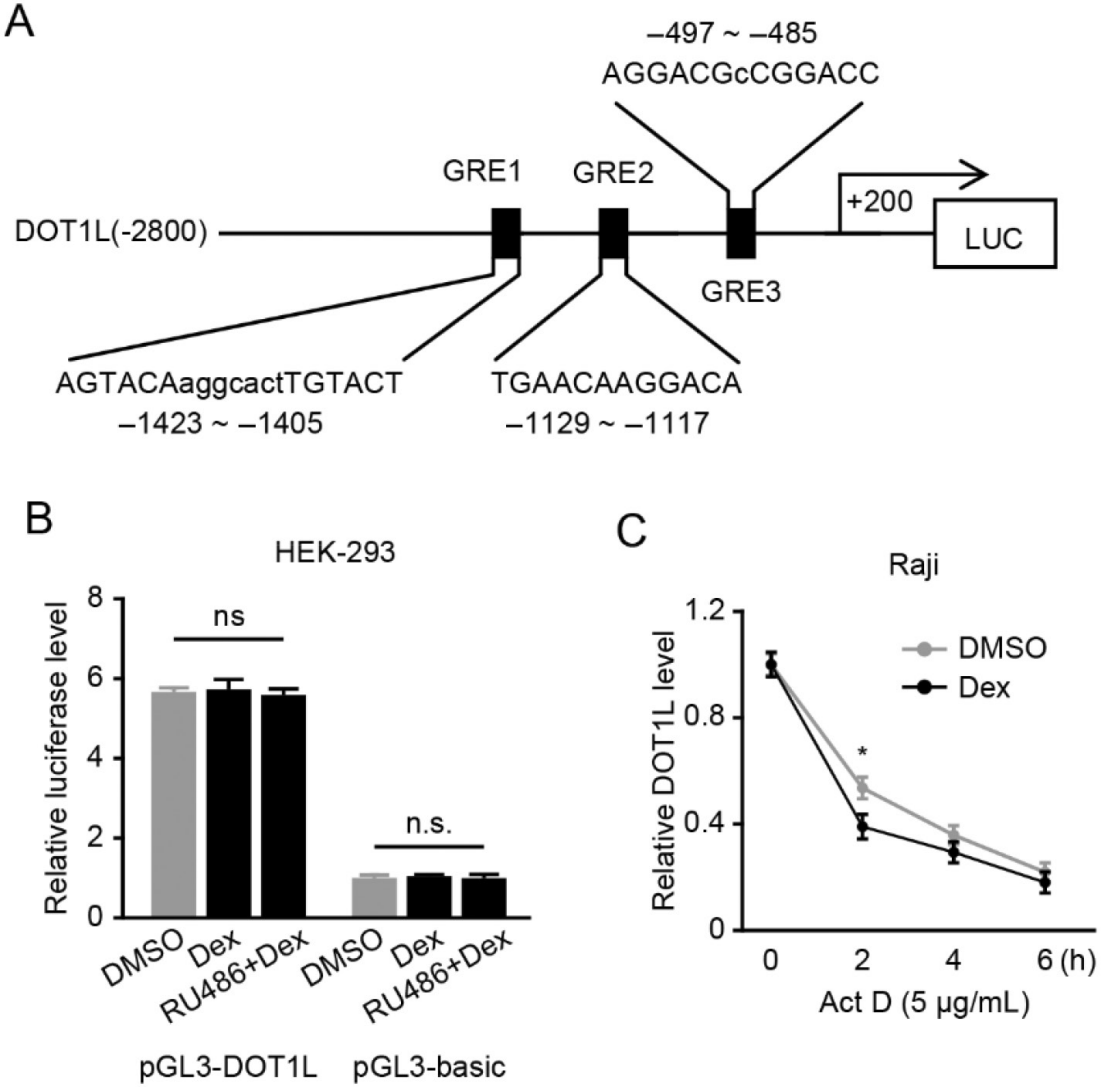
Dex decreases the mRNA level of DOT1L at the posttranscriptional level. (A) Outline of the *DOT1L* promoter region (−2800 to +200 bp) containing the putative GR‐binding sites. The −1800 to +200 bp region was fused with the pGL3‐Basic vector to obtain pGL3‐DOT1L. (B) Cells were transfected with pGL3‐DOT1L or pGL3‐Basic (control vector) in the presence of DMSO, then treated with Dex or RU486, and the Dual‐Luciferas Reporter System was used to detect the relative luciferase activity. (C) Raji cells were incubated with Act D in the presence or absence of Dex for the indicated times, and the qPCR was utilized to detect the mRNA level of *DOT1L*.

Since Dex exerts its antitumor effects primarily by activating the GR which generally functions as a transcriptional factor [21], we next investigated whether *DOT1L* was a novel target gene of GR. By bioinformatics, we found several potential GR‐binding sites in the −1800 to +200 bp of the *DOT1L* gene promoter region (−2800 to +200 bp) (Figure 4A). The fragment containing the −1800 to +200 bp region was fused with the pGL3‐Basic vector to generate pGL3‐DOT1L. The reporter assay revealed a significantly higher luciferase activity of pGL3‐DOT1L compared to that of pGL3‐Basic (Figure 4B). However, Dex did not decrease the luciferase activity of pGL3‐DOT1L, suggesting that Dex had no impact on the transcriptional activity of the *DOT1L* gene promoter. In addition, RU486 did not influence the luciferase activity. Next, we explored whether that Dex weakened the mRNA level of *DOT1L* occurred at the posttranscriptional level. As illustrated in Figure 4C, treatment with Act D, the transcriptional inhibitor, significantly decreased the mRNA level of *DOT1L*, which could be further reduced by Dex. These results suggested that Dex downregulated *DOT1L* at the posttranscriptional level.

## 4 Discussion

The present study provided the initial evidence that DOT1L was required for the proper proliferation of B‐lymphoma cells, and Dex could downregulate DOT1L expression. Such a downregulation role may be a novel mechanism for Dex to treat B lymphoma.

Aberrant posttranslational modifications play a pivotal role in cancer biology and cancer therapy [22, 23, 24, 25], one of which is the histone methylation involved in the cell cycle and somatic reprogramming [26, 27, 28]. DOT1L is the sole methyltransferase capable of catalyzing the methylation of histone H3 at lysine 79 which is considered to be involved in the development of plenty of tumors. For instance, hypermethylation of H3K79 by DOT1L is crucial for the onset of MLL‐rearranged leukemia [29] and a high level of DOT1L serves as the marker of poor prognosis in renal clear cell carcinoma [30] and ovarian cancer cells [15]. Downregulation of *DOT1L* induces a G1 arrest and cellular senescence in lung cancer cells [13] and inhibition of DOT1L suppresses the proliferation, self‐renewal, and metastasis of breast cancer cells [14]. The above evidence demonstrates that DOT1L may be a novel therapeutic target in cancer treatment. This study showed that *DOT1L* was highly expressed in B‐lymphoma cells and silencing *DOT1L* inhibited the growth of B‐lymphoma cells. To date, this is the first report to reveal the oncogenic role of DOT1L in lymphoma. This study also has some limitations. For example, the basel H3K79 expression level in lymphoma cells was merely measured by Western blot, more experiments would be useful to enhance the findings. At the same time, how DOT1L promotes the growth of B‐lymphoma cell needs further study.

Dex plays a central role in B‐lymphoma therapy and it commonly displays its anticancer efficacy via activating GR [4, 31]. GR functions mainly in three manners: first, GR binds directly to DNA to regulate gene expression, such as GR binding to the GRE of SARI promoter sequence to upregulate its mRNA level [32]; Second, GR is tethered to other transcription factors such as STAT3 and NF‐κB to affect gene expression; Third, GR binds to DNA and then they coordinate with adjacent DNA‐binding transcription factors [33]. In our study, we found that Dex downregulated *DOT1L* in a GR‐dependent manner. However, *DOT1L* was not a direct target gene of GR because GR did not suppress the activity of the *DOT1L* promoter region (−1800 to +200 bp). Furthermore, we found that Dex reduced the mRNA level of *DOT1L* at the posttranscriptional level. Since RNA‐binding proteins or microRNAs play a significant role in regulating gene expression at the posttranscriptional level [34, 35, 36], the relevant RNA‐binding proteins or microRNAs that may mediate the effect of Dex on *DOT1L* requires further investigation.

Most researchers have concentrated on the way that protein–protein interactions influence DOT1L activity; however, the upstream mechanisms that regulate DOT1L remain largely unknown. A recent study reported that CBP stabilized DOT1L at the protein level by inducing DOT1L acetylation to facilitate CRC progression and metastasis [37]. Another study showed that N‐Myc bonds to the promoter region of *DOT1L* to upregulate *DOT1L*. Silencing *DOT1L* decreased the expression of *OCD1* and *E2F2* (two target genes of N‐Myc) and suppressed the growth of neuroblastoma cells [16]. Our study showed that GR, an important transcription factor, was a novel upstream regulator of *DOT1L*. Interestingly, Myc rearrangement plays a pivotal role in the B‐lymphoma cells [38, 39]. It is of great interest to explore whether Myc rearrangement can link to the high levels of *DOT1L* in B‐lymphoma cells. On the other hand, whether Dex regulates *DOT1L* expression in MLL‐rearranged leukemia cells has aroused our great interest.

In conclusion, our study revealed that *DOT1L* is an oncogene even a new marker for therapy in B‐lymphoma cells. Downregulation of DOT1L expression by Dex may be an important mechanism for Dex to kill B‐lymphoma cells. Therefore, inhibition of DOT1L/H3K79 could be novel probes for clinically useful therapeutics in B lymphoma.

## Author Contributions

**Yuting Wang:** formal analysis, writing – original draft, investigation. **Nan Zhang:** formal analysis, investigation. **Weilong Shang:** data curation. **Huagang Peng:** data curation. **Zhen Hu:** resources. **Yi Yang:** resources. **Li Tan:** resources. **Li Zhang:** conceptualization, funding acquisition. **Fengtian He:** conceptualization, project administration, writing – review and editing. **Xiancai Rao:** writing – review and editing, conceptualization, funding acquisition, supervision.

## Conflicts of Interest

The authors declare no conflicts of interest.

## Supporting information


Figure S1.

## Data Availability

The data that support the findings of this study are available from the corresponding author upon reasonable request.
