# Supplementary material for: Dexamethasone Inhibits the Growth of B‐Lymphoma Cells by Downregulating DOT1L
Source: Cancer Rep (Hoboken). 2024 Sep 22;7(9):e2150. doi: 10.1002/cnr2.2150 (PMC11417011; doi:10.1002/cnr2.2150)
Supplement: Supplementary file 1 — Figure S1. [file CNR2-7-e2150-s001.docx]

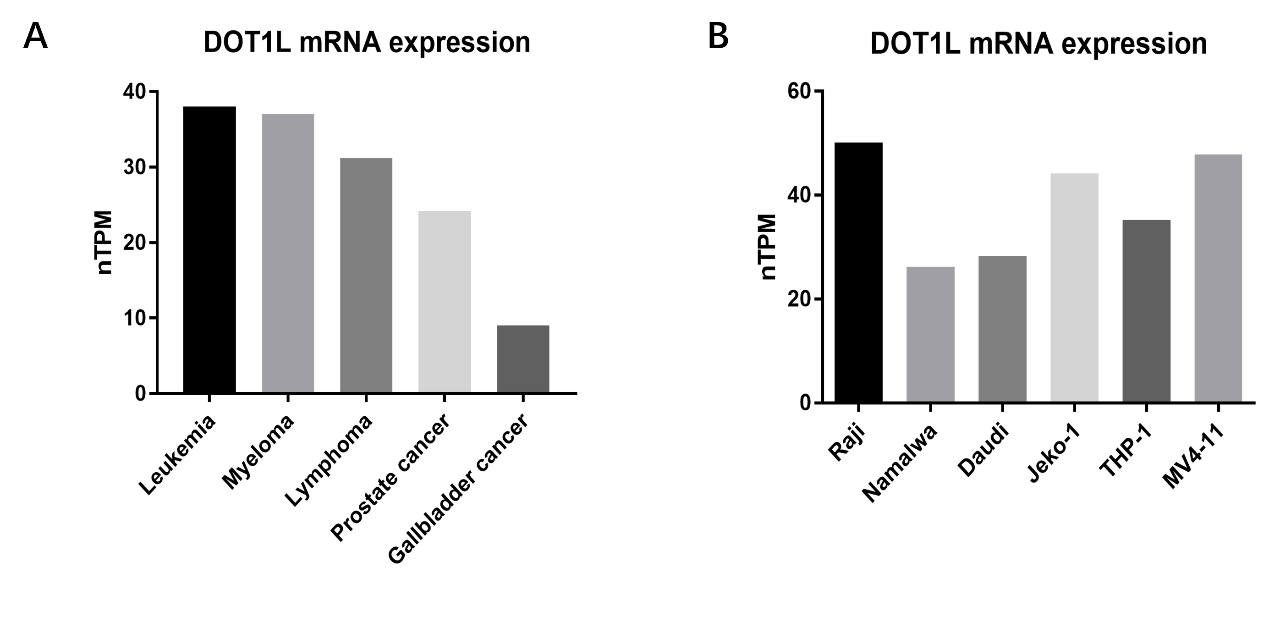


**Figure S1. DOT1L mRNA expression in various cancer cell types (A) and cell lines(B). Data were obtained from Human Protein Atlas Dataset available from**[**proteinatlas.org**](https://link.zhihu.com/?target=http%3A//proteinatlas.org/)**.**
